# Atherogenic Index of Plasma Relationship with Cardiovascular Risk Factors and Frailty and Value as Determinant of Mortality in Elderly Patients with Severe Aortic Stenosis

**DOI:** 10.3390/metabo16050289

**Published:** 2026-04-22

**Authors:** Annamaria Mazzone, Melania Gaggini, Cristina Vassalle

**Affiliations:** 1 Diagnostic and Interventional Cardiology Department, Fondazione Toscana Gabriele Monasterio, 54100 Massa, Italy; annamaria.mazzone59@gmail.com; 2 CNR Institute of Clinical Physiology, 56124 Pisa, Italy; melania.gaggini@cnr.it; 3 Fondazione Toscana Gabriele Monasterio, 56124 Pisa, Italy

**Keywords:** elderly, aortic stenosis, frailty, inflammation, physical dysfunction, mortality, atherogenic index of plasma

## Abstract

**Background**: Frailty is a common finding in elderly subjects with severe aortic stenosis (AoS) and a strong predictor of mortality and disability after aortic valve surgery. The atherogenic index of plasma (AIP) is related to different cardiovascular (CV) risk factors, which in turn are correlated to the progression of frailty as well as of AoS. **Aim**: to analyze the association of AIP with different CV risk factors and frailty scores and its value as a determinant of mortality in older adults with severe AoS. **Methods**: The association of AIP with a multidimensional assessment of frailty by using Fried criteria and the following indices; timed up-and-go test (TUG) for gait function; Charlson Index (CI), basic activities of daily living (BADL) and instrumental activities of daily living (IADL) for disability; mini–mental state examination for cognitive function evaluation (MMSE); Geriatric Depression Score for mood disorder (GDS); Mini Nutritional Assessment (MNA) for nutritional status was assessed in 102 elderly AoS patients (33 males; mean age 83 ± 6 yrs). Moreover, the relationship between AIP and demographic, lifestyle, traditional CV risk factors and CV mortality was also evaluated. **Results**: Significant relationships between AIP and glycemia and inflammatory parameters (CRP, ESR and fibrinogen) as well as with troponin I were found. Moreover, AIP significantly correlates with CI, BADL, IADL and MNA. However, the Kaplan–Meier analysis did not show any significant difference for survival rates according to AIP intervals of risk, whereas ejection fraction remained the only significant determinant after multivariate adjustment for mortality at the Cox proportional hazard models analysis in this patient population. **Conclusions**: Higher AIP is significantly associated with cardiometabolic risk and increased physical dysfunction risk and frailty in AoS pts, evidencing its potential use as a simple biomarker in this clinical setting, although it did not represent a significant determinant for mortality in this population.

## 1. Introduction

Aortic stenosis (AoS) is not a passive process of calcium deposition that occurs with advanced age, but rather an active metabolic and inflammatory disease, closely related to inflammaging (a chronic low-grade systemic inflammation related to aging) and metabolic dysfunction [1]. This condition is characterized by processes also common to the development of atherosclerotic damage, involving oxidized lipids, T lymphocytes, and monocytes as main inflammatory features that contribute to valvular fibrosis and calcification processes; in this context, metabolic risk factors (e.g., obesity, Type 2 diabetes-T2D- and lipid dysfunction) that accelerate atherosclerosis, also facilitate the fibrocalcific remodeling [2]. Inflammaging in particular represents a key driver linking pathophysiological processes associated with aging and cardiometabolic diseases (e.g., AoS, frailty, atherosclerosis, obesity, dyslipidemia and T2D) and fuels a vicious cycle driving the development of structural cardiovascular diseases as well as degenerative AoS [3].

AoS is a common valvular disease, especially among elderly subjects [4]. Surgical options represent the only definitive treatments (either aortic valve replacement-SAVR- or transcatheter aortic valve replacement-TAVR, which is less invasive and more often used in patients > 70 years, with comorbidities and a higher surgical risk), as no medical treatment has been shown to be reliable to regress or prevent the calcification process [4,5]. Nonetheless, a significant percentage of patients may not benefit from surgery, as they do not show any advantage in terms of mortality risk or quality of life improvement, highlighting the importance of establishing preliminary criteria to avoid inappropriate interventions in such patients [6,7]. In this context, frailty, which implies increased physical and cognitive disability, is a common finding in elderly AoS patients and a strong predictor of mortality and disability after aortic valve surgery [8]. However, at present, the evaluation of frailty remains challenging, as there is no consensus on the exact definition or on gold standard biomarkers to use, leading to delays in diagnosis, subjectivity, and a lack of uniformity according to domains considered. As no single definition of frailty is exhaustive and used as the primary parameter of frailty reference, frailty is better described using a multidimensional assessment, which may include evaluation of physical function, cognition, depression, nutrition and medical comorbidities [9].

Clearly, given the great interest in early detection and prevention/treatment of frailty and functional decline, which can reduce adverse events in older individuals (also in the AoS population), further data in this field may provide new insights on the pathophysiology of these processes, helping to identify additional easily measurable biomarkers for risk assessment and personalized interventions and allowing more tailored therapeutic strategies for specific profiles derived by key biomarker modulation, such as lipids. In this context, lipid profiling may be significant because dyslipidemia supports frailty development, promoting chronic inflammation, visceral obesity, and metabolic abnormalities and lipid alterations have been found associated with reduced physical function and muscle weakness in elderly subjects [10]. Accordingly, the atherogenic index of plasma (AIP), calculated as the logarithm of the ratio of triglycerides to high-density lipoprotein cholesterol (TG/HDL-C), initially proposed as an additive biomarker to assess dyslipidemia, has demonstrated a close relationship with cardiometabolic risk, and atherosclerosis, with oxidative stress, inflammation, and insulin resistance as key underlying mechanisms related to its adverse effects [11,12,13]. In turn, all these risk factors and events are closely related to the progression of frailty domains as well as of AoS [14,15]. However, although recent emerging evidence shows how high AIP levels are related to functional impairment and disability, no data are available regarding specific frailty scores as well as in a multi-dimensional assessment of frailty and in the elderly population with AoS [16,17]. Interestingly, AIP presents unique and interesting characteristics when compared to other lipid-related scores in the evaluation of CV risks, as it reflects lipid abnormalities related to TG and HDL-C, quantitatively estimating the balance between atherogenic and anti-atherogenic lipoprotein fractions. For these reasons, we chose this index to investigate in the present study its relationship with a multidimensional assessment of physical dysfunction and frailty in elderly AoS patients.

## 2. Materials and Methods

### 2.1. Patient Population

Data from a total of 102 elderly patients (33 males; 83 ± 6 years) afferent to the Ospedale del Cuore FTGM in Massa, Italy (1 March 2016–30 March 2020), for a multidimensional assessment including clinical, laboratory, surgical, and geriatric risk evaluation for tailored treatment of valve disease (SAVR, TAVR, balloon aortic valvuloplasty (BAV), or medical therapy (MT)), followed during a mean follow-up period of 18 ± 5 months, were retrospectively analyzed [18].

The inclusion criteria were: (1) a diagnosis of severe AoS (patients with an effective orifice area < 1 cm^2^ and/or <0.6 cm^2^/m^2^ body surface area according to the AHA/ACC Guideline for the Management of Patients with Valvular Heart Disease) of degenerative etiology [19]; (2) preserved systolic function (3) symptoms (dyspnea, angina, syncope); (4) age 75 years or older; (5) a written informed consent. The exclusion criteria were: (1) acute heart failure, (2) hemodynamic shock or (3) the presence of active infection at the time of patient enrolment, due to the potential impact upon the results of the biomarkers evaluated.

Medical therapy included angiotensin-converting enzyme inhibitors, beta-blockers, calcium antagonists, MRA/ARA, lipid-lowering agents, antidiabetic agents, diuretics, and oral anticoagulant therapy; all patients had polypharmacy (mean number of drugs: 7 ± 2.5).

The study was approved by the local Ethics Committee (No. 22239).

### 2.2. Assessment of Frailty

Patients underwent a clinical, laboratory, and multi-geriatric assessment (MGA) to evaluate comorbidities, disability, cognitive function, depression, and nutritional status, using the following validated parameters (Table 1): (1) Charlson Index (CI) with a cut-off value of >2 for comorbidity [20]; (2) basic activities of daily living (BADL) and instrumental activities of daily living (IADL) for disability [21]; (3) mini–mental state examination for cognitive function evaluation (MMSE at 18 points, cognitive impairment) [22]; (4) Geriatric Depression Score for mood disorder (GDS over 5 points, depression) [23]; (5) Mini Nutritional Assessment (MNA for nutritional status) [24]. Moreover, the identification of frailty was identified by using the phenotypic Fried criteria and categorized as robust or pre-frail = 0–2, frail = 3–5 [25]. We also considered the following parameters: slowness, using the timed up-and-go test (TUG) for gait function (TUG at 20 s) [26]. Moreover, the STS (Society of Thoracic Surgeons) score for aortic stenosis was calculated to estimate the risk of death from aortic valve replacement surgery or transcatheter aortic valve replacement (TAVR) in patients with aortic stenosis according to the following scale: ≥8 high, 4–8 intermediate, <4 low risk [27].

### 2.3. Echocardiographic Evaluation

All patients underwent an echocardiographic assessment to evaluate echocardiographic parameters, including aortic transvalvular mean gradient (mAVG; mmHg), left ventricular ejection fraction (EF; %), and pulmonary artery pressure (PAPs; mmHg) [19].

### 2.4. Laboratory Assessment

In all patients, a venous blood sample was withdrawn following 12 h fasting, and blood parameters measured by automatic biochemical analyzers included a complete lipid profile [total cholesterol (TC), triglycerides (TG), high-density lipoprotein cholesterol (HDL-C), triglycerides (TG), low-density lipoprotein cholesterol (LDL-C)], fasting blood glycemia, inflammatory/oxidative stress-related biomarkers [C reactive protein (CRP), uric acid, erythrocyte sedimentation rate (ESR), fibrinogen], thyroid function (thyroid-stimulating hormone), hepatic enzymes [gamma-glutamyl transpeptidase (GGT), aspartate aminotransferase (AST), alanine aminotransferase (ALT)], cardiac biomarkers [brain natriuretic peptide (BNP), troponin I]. AIP was calculated using the formula: AIP = −log10 (TG/HDL-C). Then, AIP was categorized into three risk levels as follows: values < 0.11 indicate low risk, 0.11–0.21 moderate risk, and >0.21 indicate high risk for atherosclerosis [28].

### 2.5. Statistical Methods

Continuous variables were given as mean ± SD, while categorical variables were reported using frequencies (percentages). The logarithmic transformation was used to make data closer to a normal distribution and stabilize variance for distributions skewed to the right. The comparison of AIP levels between the two groups was assessed using Student’s *t*-test for continuous variables. Linear regression was performed to assess the relationship between a continuous dependent variable (AIP) and one or more independent variables (predictors).

Cumulative event rates were estimated by Kaplan–Meier survival curves and probability values were determined with the log-rank test. Statistical analysis also included the Cox proportional hazard model to determine the value of AIP as an independent predictor of mortality.

A *p*-value < 0.05 was considered statistically significant.

## 3. Results

### 3.1. Patient Characteristics 

General and clinical characteristics of 102 participants are reported in Table 2.

**Figure 1:**
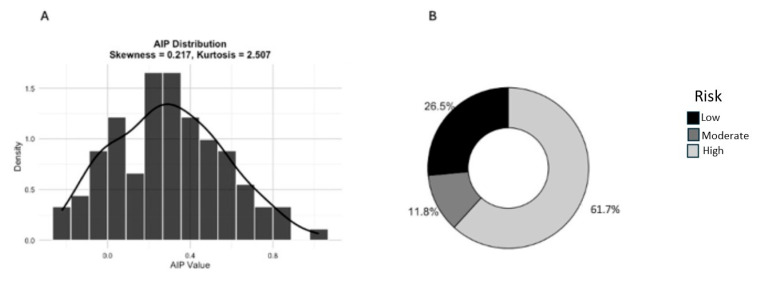
AIP distribution (panel (**A**)) and percentage of patients according to AIP risk intervals (panel (**B**)).

Mean AIP value in the overall population was 0.3 ± 0.27; AIP distribution is reported in Figure 1 (panel A) as well as the number (percentage) of patients according to AIP levels (<0.11 low risk, 0.11–0.21 moderate risk, and >0.21 high risk for atherosclerosis; panel B).

### 3.2. Relationship Between AIP and the Other Clinical and Laboratory Parameters

Simple regression analysis between AIP and the clinical and laboratory variables was performed and significant relationships are reported in Table 3.

Levels of AIP did not significantly differ between males and females (0.26 ± 0.26 vs. 0.32 ± 0.27, *p* = ns), nor with aging (R^2^ = 0.03, *p* = ns), but resulted in higher levels in T2D patients (0.4 ± 0.28 vs. 0.24 ± 0.14 in no-T2D, *p* < 0.001).

### 3.3. Relationship Between AIP and Frailty Scores

When simple regression analysis between AIP with frailty scores and STS score was performed, significant relationships (although weak) between AIP and CI, BADL, IADL and MNA were found and reported in Table 3.

### 3.4. Multiple Regression for AIP

Multiple regression analysis showed that glycemia (T-value 2.6, *p* < 0.05) and ESR (2.5, ≤0.01) remained as independent determinants affecting AIP levels in our population (Table 4).

### 3.5. Kaplan–Meier Survival Curve for Mortality According to AIP Values 

**Figure 2:**
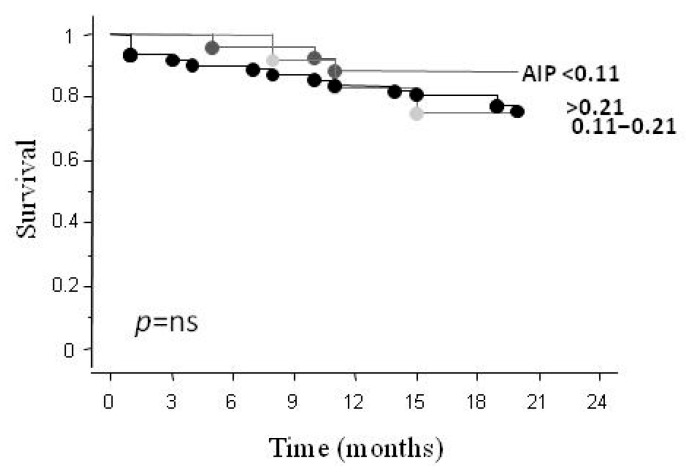
Kaplan–Meier curve for total mortality according to AIP levels.

During the follow-up period (18 ± 5 months), 21 (20%) CV deaths were recorded. The Kaplan–Meier analysis did not show any significant difference for survival rates according to AIP intervals of risk (Figure 2).

### 3.6. Determinants of Mortality at the Cox Proportional Hazard Models Analysis

AIP concentration was not found to be an independent predictor of overall mortality at the univariate Cox proportional hazard model analysis. Instead, among univariate significant parameters (EF, mAVG, PAPs, HDL, VES and CRP), EF remained the only significant determinant after multivariate adjustment for mortality in this patient population.

## 4. Discussion

High AIP is significantly associated with cardiometabolic risk and increased physical dysfunction and frailty in severe AoS pts, but it did not represent a significant determinant for mortality in this population. AIP is easily calculable by available biochemical parameters and is cost-free. Thus, its measurement (also a regular monitoring) in elderly AoS patients is feasible and can help in the early identification and implementation of tailored interventions to break and retard the cycle of decline related to cardiometabolic risk, physical dysfunction risk and frailty in this clinical setting. Moreover, AIP use might assist to better risk-stratifying SAVR/TAVR candidates on the basis of its relationship with the level of patient frailty.

From its introduction in 2000 by Dobiásová et al., AIP has emerged as a reliable biomarker to estimate lipid-related cardiovascular risk [29]. An important reason for this association is the fact that AIP, expressed by high TG and low HDL-C, reflects over other lipid parameters its strong association with a pro-atherogenic lipid profile characterized by the presence of smaller, denser, and more atherogenic particle (small dense low-density lipoprotein cholesterol sd-LDL-C), at increased atherogenic potential and pro-inflammatory and pro-oxidative, because subjected to oxidation and glycation [24]. Moreover, HDL-C, which takes part in AIP calculation, is a particle involved in the transport of peripheral cholesterol to the liver, and contains antioxidant enzymes (e.g., paraoxonase) that protect the other lipoproteins against oxidation [30]. These lipid alterations (increased TG and reduced HDL-C), which lead to increased oxidative stress driven by lipid peroxidation, may also affect mitochondrial efficiency, disrupting normal energy production (with effects on the electron transport chain and the AMPK/PGC-1α signaling pathway, a regulator of mitochondrial biogenesis and cellular energy homeostasis) [31,32]. Elevated AIP levels are also significantly correlated with systemic inflammation (e.g., CRP and cytokines, such as interleukin-6) and oxidative stress in subjects at risk or with T2D [33,34]. Insulin resistance, an index of glicometabolic dysfunction, and blood glycemia are other important biomarkers related to high AIP in other clinical settings [33,35]. Thus, as ESR also reflects systemic inflammation, higher glycemia and ESR can be associated with elevated AIP levels in conditions with combined inflammatory and metabolic dysfunction. Accordingly, a review including 32 studies and focused on the general population, evidenced that AIP levels were associated with cardiometabolic risk (e.g., waist circumference and insulin resistance) [11]. Recent data also evidence the association of AIP with adverse events in a large general population (myocardial infarction, acute coronary syndrome, stroke, heart failure and mortality; 6323 over 35-year-old healthy adults from 2001 to 2016) [28]. Moreover, a recent meta-analysis focused on patients with suspected or established coronary artery disease (CAD) showed the relationship between AIP levels and increased CAD risk, greater severity, and adverse prognosis [36]. Specifically, patients with elevated AIP were more likely to have CAD, coronary artery calcification, multivessel CAD, and an increased risk of plaque progression. AIP levels were also associated with a worse cardiovascular prognosis [36]. Accordingly, a meta-analysis including sixteen studies for a total number of 20,833 CAD patients showed AIP as consistently associated with an increased risk of major adverse cardiovascular events, cardiovascular death, myocardial infarction, revascularization, and the no-reflow phenomenon [37]. In our study, the Kaplan–Meier analysis did not show any significant difference between subjects in the low-risk AIP group (<0.11) with respect to those in the group with higher AIP levels in terms of different survival rates, and consequently, AIP did not represent an independent parameter for mortality. To note, a consistent subgroup of patients in our population was taking statins (40%), which may likely contribute to lower AIP values by reducing TG levels and increasing HDL-C; this is an important fact that may have weakened its association with mortality. Thus, the potential confounding statin roles in the relationship between AIP levels and prognosis merits to be better deepened in future studies. Moreover, for it concerns the relationship between AIP and mortality, interesting recent findings (10 years of follow-up, 2077, 262, 854 subjects, 476 cases of all-cause mortality) suggest a U-shaped association between AIP and all-cause mortality [38]. Specifically, this relationship was explained according to the fact that higher AIP predicted a higher risk of death from diabetes mortality, whereas the lowest AIP predicted a higher risk of death from non-diabetes mortality [38]. Effectively, a low AIP may reflect malnutrition or metabolic derangements, thereby predisposing to an adverse outcome [39]. In our specific population, mortality at lower AIP levels might be related, almost in part, to malnutrition; accordingly, we previously showed that malnutrition (hypoalbuminemia) makes patients more vulnerable to poorer outcomes after cardiovascular procedures and represented one key determinant of mortality in this kind of patient [18]. Interestingly, when a multivariate adjustment was done to assess the AIP association with frailty domains significant at the univariate analysis (BADL, IADL, MNA and CI) and key frailty parameter as age, gender, BNP, creatinine and EF, a significant relationship remains with BADL (T-value −2.1, *p* < 0.05) and MNA (T-value −2.1, *p* < 0.05), which seems to confirm the relationship between AIP and malnutrition also in our population.

Different results have linked AIP to frailty-related outcomes, primarily sarcopenia [40,41]. However, seeing the complexity, no specific definition can be used as the primary outcome. Thus, as neither examined frailty as a multidimensional reality nor evaluated AIP’s predictive role in AoS patients, this aspect is the strength of our study. There are also limitations. This was a single-center study with a relatively small sample size. To address this aspect, when a post hoc power analysis was performed (G*Power 3.1 program), a study power (1 − β) of 0.99 was obtained, considering a medium effect size of 0.5 and an α value of 0.05. Unfortunately, the measurement of lipoprotein(a), evidenced in 2022 by the European Atherosclerosis Society consensus statement as one key risk factor for aortic valve stenosis, was not available in our population [42]. However, as aortic stenosis is in turn closely linked to the development of frailty in the elderly, it would be interesting to include this lipid biomarker in future studies. Moreover, it would be interesting to further evaluate present relationships in other cohorts and other clinical settings.

The association between AIP and frailty, as confirmed by our study, may be due to different underlying biological interrelated aspects that connect these two parameters. Specifically, by integrating key lipid biomarkers into a single score, AIP contributes to a more comprehensive evaluation of cardiometabolic risk and inflammatory alterations, aligning with the multifactorial nature of frailty. Thus, our results suggest the possibility of integrating AIP, easily obtainable from standard lipid panels, into routine elderly AoS patient assessment to identify and manage frailty, implying possible clinical application. In fact, this approach not only may improve the knowledge of lipid-related risk burden but also favors the development of early and more tailored and effective strategies to face the risk of long-term disability (e.g., by acting on lipid modulation and lifestyle habits), delaying or even preventing the effects of frailty advancement.

## 5. Conclusions

AIP is related to cardiometabolic risk and frailty in elderly AoS patients, thus representing a holistic biomarker for these conditions, especially when considering its availability and cost-effectiveness, which make it a potentially interesting complement to other tools in both clinical and research settings.

## Figures and Tables

**Table 1:** Multidimensional evaluation to assess frailty and STS score.

Index	Acronym	Criteria	Mean Values in the Studied Population	Interpretation	Ref.
Charlson Index	CI	Prediction of ten-year mortality for a patient with comorbidities	4 ± 2	≥2 high-risk of mortality	[20]
Basic activities of daily living	BADL	Fundamental self-care tasks to manage personal needs	5 ± 1	0 (unable) to 6 (maximal function)	[21]
Instrumental activities of daily living	IADL	Capacity to perform complex tasks necessary for independent living	6 ± 2	Score ranging from 0 (fully dependent) to 8 (fully independent)	[21]
Mini–mental state examination for cognitive function evaluation	MMSE	To assess cognitive impairment (five areas of cognition: orientation to time and place, attention and calculation, recall, language, and drawing, with scores influenced by factors like age, education, and language skills)	24 ± 6	≤18 severe cognitive impairment	[22]
Geriatric Depression Score for mood disorder	GDS	A total score range of 0 to 15, where higher scores reflect more severe levels of depression.	3.7 ± 3.2	Scores of 6–10 indicate mild to moderate depression, while scores of 11–15 indicate severe depression.	[23]
Mini Nutritional Assessment	MNA	0–14 points	10 ± 2	<8 points (malnutrition)	[24]
Fried Phenotypic Score	FRIED	- Weight loss, exhaustion - Low physical activity - Slow walking speed - Weakness (grip strength)	3 ± 1	Pre-frail = 1–2, frail = 3–5 criteria	[25]
Timed up-and-go test	TUG	Time to stand up from a chair, walk a short distance, turn around, and sit back down	24 ± 10	<20 s (normal gait function) ≥20 s (mobility impairment)	[26]
Society of Thoracic Surgeons score	STS	Risk of death from aortic valve surgery in AoS patients	5 ± 5	≥8 high, 4–8 intermediate, <4 low risk	[27]

**Table 2:** Characteristics of the patient population.

Anthropometric and Clinical Parameters	Values
Number	102
Age (yrs)	83 ± 6
Sex (M/F)	33/69 (22/68)
Hypertension	74 (72)
T2D	37 (36)
Dyslipidemia	74 (72)
Smoking	29 (29)
COPD	42 (41)
Previous ischemic event	31 (30)
Chronic heart failure	65 (64)
**Echocardiographic** **Parameters**	
Pulmonary artery systolic pressure (PAPs; mmHg)	47 ± 11
Mean aortic valve gradient (mAVG; mmHg)	44 ± 13
Left ventricular ejection fraction (EF; %)	58 ± 9
Septum (mm)	13.7 ± 1.9
E/A ratio (≤1)	80 (78)
E/A ratio (>1)	22 (22)
Left atrial area (cm^2^)	26 ± 7
Left Ventricular End-Diastolic Diameter (mm)	49 ± 7
Left Ventricular End-Systolic Diameter (mm)	30 ± 7
**Biochemical Parameter**	
Glucose (mg/dL)	116 ± 34
Total cholesterol (mg/dL)	185 ± 36
Triglycerides (mg/dL)	121 ± 66
HDL-C (mg/dL)	56 ± 16
LDL-C (mg/dL)	106 ± 31
CRP (mg/dL)	0.7 ± 1.5
ESR (mm/h)	38 ± 23
Fibrinogen (mg/dL)	377 ± 118
Uric acid (mg/dL)	6.1 ± 1.9
TSH (mIU/L)	2.5 ± 7
GGT (UI/L)	31 ± 36
AST (UI/L)	22 ± 10
ALT (UI/L)	16 ± 10
Troponin I (ng/L)	0.032 ± 0.035
BNP (pg/mL)	555 ± 948

Data are mean ± DS or number (%) T2D = type 2 diabetes; COPD = chronic obstructive pulmonary disease; HDL-C = High-Density Lipoprotein Cholesterol; LDL-C = Low Density Lipoprotein Cholesterol; CRP = C-reactive protein; ERS = Erythrocyte Sedimentation Rate; TSH = Thyroid-Stimulating Hormone; GGT = Gamma-Glutamyl Transpeptidase; AST = Aspartate aminotransferase; ALT = alanine aminotransferase; BNP = Brain Natriuretic Peptide.

**Table 3:** Simple regression between AIP and laboratory markers and frailty scores.

Parameter	R^2^	*p*
log (Glucose)	0.15	<0.001
log (CRP)	0.1	<0.01
log (ESR)	0.21	<0.001
log (Fibrinogen)	0.15	<0.001
Creatinine	0.05	<0.05
CI	0.05	<0.05
BADL	0.06	<0.05
IADL	0.04	≤0.05
MNA	0.1	<0.01

R^2^ = coefficient of determination; *p* = *p*-value.

**Table 4:** Multiple regression between AIP and different risk factors.

Variable	Standard Coefficient	t-Value	*p*
log (Glucose)	0.25	2.6	<0.05
log (CRP)	0.03	0.23	ns
log (ESR)	0.33	2.5	≤0.01
log (Fibrinogen)	0.06	0.4	ns
Creatinine	0.06	0.6	ns
CI	0.02	0.16	ns
BADL	−0.16	−1.1	ns
IADL	0.08	0.52	ns
MNA	−0.05	−0.5	ns

*p* = *p*-value.

## Data Availability

The original contributions presented in this study are included in the article. Further inquiries can be directed to the corresponding author.
